# 2,3,4-Tri-*O*-acetyl-β-l-arabinopyranosyl trichloro­acetimidate

**DOI:** 10.1107/S1600536811002005

**Published:** 2011-01-22

**Authors:** Zu-Li Sun

**Affiliations:** a Science and Engineering College of Chemistry and Biology, Yantai University, Yantai 264005, People’s Republic of China

## Abstract

In the title compound, C_13_H_16_Cl_3_NO_8_, the trichloro­acetimidate group is located in an axial postion on the anomeric carbon of the sugar ring.

## Related literature

For applications of glycosyl trichloro­acetimidate in glycosyl bond formation, see: Schmidt & Zhu (2008 ▶). For the preparation of the title compound, see: Schmidt & Stumpp (1983 ▶).
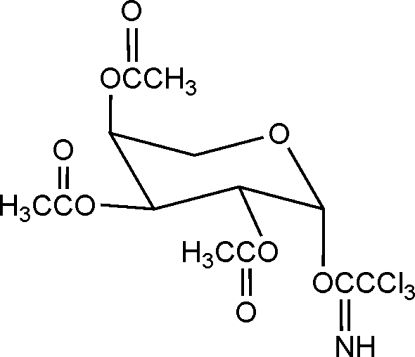


## Experimental

### 

#### Crystal data

C_13_H_16_Cl_3_NO_8_
*M* *_r_* = 420.62
Monoclinic, 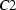

*a* = 21.384 (5) Å
*b* = 6.7994 (16) Å
*c* = 13.096 (3) Å
β = 96.796 (3)°
*V* = 1890.7 (8) Å^3^
*Z* = 4
Mo *K*α radiation
μ = 0.52 mm^−1^
*T* = 298 K
0.49 × 0.24 × 0.08 mm

#### Data collection

Bruker SMART CCD area-detector diffractometer
Absorption correction: multi-scan (*SADABS*; Bruker, 2003 ▶) *T*_min_ = 0.784, *T*_max_ = 0.959
4972 measured reflections
3239 independent reflections
2933 reflections with *I* > 2σ(*I*)
*R*_int_ = 0.021

#### Refinement

*R*[*F* ^2^ > 2σ(*F* ^2^)] = 0.037
*wR*(*F* ^2^) = 0.087
*S* = 1.06
3239 reflections
226 parameters
1 restraint
H-atom parameters constrained
Δρ_max_ = 0.23 e Å^−3^
Δρ_min_ = −0.17 e Å^−3^
Absolute structure: Flack (1983 ▶), 1334 Friedel pairs
Flack parameter: 0.04 (6)

### 

Data collection: *SMART* (Bruker, 2003 ▶); cell refinement: *SAINT* (Bruker, 2003 ▶); data reduction: *SAINT*; program(s) used to solve structure: *SHELXS97* (Sheldrick, 2008 ▶); program(s) used to refine structure: *SHELXL97* (Sheldrick, 2008 ▶); molecular graphics: *SHELXTL* (Sheldrick, 2008 ▶); software used to prepare material for publication: *SHELXTL*.

## Supplementary Material

Crystal structure: contains datablocks global, I. DOI: 10.1107/S1600536811002005/jh2255sup1.cif

Structure factors: contains datablocks I. DOI: 10.1107/S1600536811002005/jh2255Isup2.hkl

Additional supplementary materials:  crystallographic information; 3D view; checkCIF report

## Figures and Tables

**Fig. 1.**
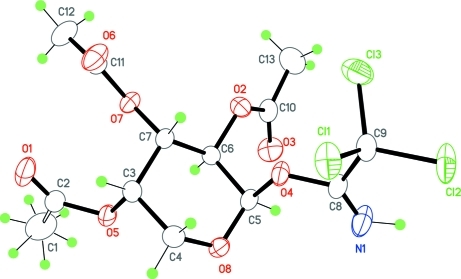
Elipsoid plot.

## supplementary crystallographic information

### Comment

The title compound, a very useful glycosyl intermediate in glycosyl bond formation, was prepared from L-arabinose according to literature (Schmidt *et al.*, 1983) and its structure was further characterized by X-ray crystallographic techniques. The structure of 2,3,4-tri-*O*-acetyl-β-L-arabinopyranosyl trichloroacetimidate has monoclinic (C2) symmetry. The structure reveals that the trichloroacetimidate group is located at axial postion on anomeric carbon of sugar ring, as shown in Fig. 1. There is hydrogen bond interaction between N (1) and Cl (2) on the trichloroacetimidate group [N(1)···Cl(2) 3.009 (3) Å and N(1)—H(1)···Cl(2) 113.5 °].

### Experimental

The title compound was prepared from L-arabinose by the following three steps: i) acetylation with Ac_2_O in pyridine; ii) selective removal of acetyl group on anomeric carbon by ammonia methanol solution; iii) trichloroacetimidate formation with Cl_3_CN/DBU in dichloromethane. Colorless single crystals were grown from a solution of petroleum ether/ethyl acetate (2:1).

### Refinement

All non-hydrogen atoms were refined with anisotropic displacement parameters. Hydrogen atoms attached to anisotropically refined atoms were placed in geometrically idealized positions and included as riding atoms with C—H = 0.98Å and *U*_iso_(H) = 1.2**U*_eq_(C) (–CH); C—H = 0.96Å and *U*_iso_(H) = 1.5**U*_eq_(C) (methyl); C—H = 0.97Å and *U*_iso_(H) = 1.2**U*_eq_(C) (methylene); N—H = 0.88Å and *U*_iso_(H) = 1.2**U*_eq_(N). The H atoms of one methyl is disordered over two closely spaced positions in 0.5/0.5 ratio.

### Crystal data

**Table:**

C_13_H_16_Cl_3_NO_8_	*F*(000) = 864
*M**_r_* = 420.62	*D*_x_ = 1.478 Mg m^−^^3^
Monoclinic, *C*2	Mo *K*α radiation, λ = 0.71073 Å
*a* = 21.384 (5) Å	Cell parameters from 2245 reflections
*b* = 6.7994 (16) Å	θ = 2.6–23.4°
*c* = 13.096 (3) Å	µ = 0.52 mm^−^^1^
β = 96.796 (3)°	*T* = 298 K
*V* = 1890.7 (8) Å^3^	Plan, colourless
*Z* = 4	0.49 × 0.24 × 0.08 mm

### Data collection

**Table:**

Bruker SMART CCD area-detector diffractometer	3239 independent reflections
Radiation source: fine-focus sealed tube	2933 reflections with *I* > 2σ(*I*)
graphite	*R*_int_ = 0.021
φ and ω scans	θ_max_ = 25.5°, θ_min_ = 1.9°
Absorption correction: multi-scan (*SADABS*; Bruker, 2003)	*h* = −25→21
*T*_min_ = 0.784, *T*_max_ = 0.959	*k* = −8→6
4972 measured reflections	*l* = −12→15

### Refinement

**Table:**

Refinement on *F*^2^	Secondary atom site location: difference Fourier map
Least-squares matrix: full	Hydrogen site location: inferred from neighbouring sites
*R*[*F*^2^ > 2σ(*F*^2^)] = 0.037	H-atom parameters constrained
*wR*(*F*^2^) = 0.087	*w* = 1/[σ^2^(*F*_o_^2^) + (0.0276*P*)^2^ + 0.9525*P*] where *P* = (*F*_o_^2^ + 2*F*_c_^2^)/3
*S* = 1.06	(Δ/σ)_max_ = 0.006
3239 reflections	Δρ_max_ = 0.23 e Å^−^^3^
226 parameters	Δρ_min_ = −0.17 e Å^−^^3^
1 restraint	Absolute structure: Flack (1983), 1334 Friedel pairs
Primary atom site location: structure-invariant direct methods	Flack parameter: 0.04 (6)

### Fractional atomic coordinates and isotropic or equivalent isotropic displacement parameters (Å^2^)

**Table:**

	*x*	*y*	*z*	*U*_iso_*/*U*_eq_	Occ. (<1)
C2	0.61191 (15)	0.4264 (8)	0.4040 (2)	0.0721 (11)	
C3	0.71252 (13)	0.5071 (4)	0.3556 (2)	0.0431 (7)	
H3A	0.6937	0.6380	0.3448	0.052*	
C4	0.77597 (14)	0.5222 (4)	0.4187 (2)	0.0479 (7)	
H4A	0.8017	0.6154	0.3862	0.057*	
H4B	0.7704	0.5729	0.4863	0.057*	
C5	0.81676 (12)	0.2479 (4)	0.33786 (19)	0.0385 (6)	
H5A	0.8369	0.1197	0.3519	0.046*	
C6	0.75450 (12)	0.2193 (4)	0.27061 (18)	0.0354 (6)	
H6A	0.7285	0.1266	0.3044	0.042*	
C7	0.71942 (11)	0.4112 (4)	0.25338 (17)	0.0354 (5)	
H7A	0.7423	0.4991	0.2115	0.042*	
C8	0.91695 (12)	0.3220 (4)	0.2839 (2)	0.0449 (7)	
C9	0.94598 (13)	0.4576 (5)	0.2087 (2)	0.0518 (7)	
C10	0.77615 (13)	−0.0517 (4)	0.1675 (2)	0.0453 (7)	
C11	0.62909 (13)	0.5066 (5)	0.1420 (2)	0.0461 (7)	
C12	0.56681 (14)	0.4375 (7)	0.0926 (3)	0.0725 (10)	
H12A	0.5464	0.5418	0.0519	0.109*	
H12B	0.5412	0.3995	0.1447	0.109*	
H12C	0.5726	0.3267	0.0493	0.109*	
C13	0.78124 (19)	−0.1138 (5)	0.0615 (3)	0.0723 (10)	
H13A	0.7903	−0.2520	0.0604	0.109*	
H13B	0.8145	−0.0419	0.0353	0.109*	
H13C	0.7422	−0.0881	0.0195	0.109*	
Cl1	0.93304 (4)	0.70622 (13)	0.24083 (7)	0.0688 (3)	
Cl2	1.02723 (4)	0.41909 (16)	0.21284 (8)	0.0838 (3)	
Cl3	0.90994 (5)	0.40977 (19)	0.08308 (6)	0.0833 (3)	
O1	0.58775 (12)	0.5583 (6)	0.3561 (2)	0.0997 (11)	
O2	0.76398 (9)	0.1433 (2)	0.17167 (13)	0.0429 (4)	
O3	0.78190 (11)	−0.1520 (3)	0.24214 (18)	0.0617 (6)	
O4	0.85537 (8)	0.3662 (3)	0.27922 (14)	0.0441 (4)	
O5	0.67371 (8)	0.3848 (3)	0.41230 (13)	0.0521 (5)	
O6	0.65015 (10)	0.6661 (3)	0.13335 (17)	0.0622 (6)	
O7	0.65915 (8)	0.3634 (3)	0.19901 (13)	0.0436 (5)	
O8	0.80828 (8)	0.3379 (3)	0.43005 (13)	0.0445 (5)	
N1	0.94316 (12)	0.1918 (5)	0.3390 (2)	0.0720 (9)	
H1	0.9834	0.1736	0.3329	0.108*	
C1	0.57884 (18)	0.2786 (11)	0.4638 (3)	0.125 (2)	
H1A	0.5975	0.2786	0.5342	0.187*	0.50
H1B	0.5828	0.1502	0.4349	0.187*	0.50
H1C	0.5351	0.3126	0.4607	0.187*	0.50
H1'1	0.5638	0.3421	0.5217	0.187*	0.50
H1'2	0.6077	0.1757	0.4877	0.187*	0.50
H1'3	0.5439	0.2236	0.4204	0.187*	0.50

### Atomic displacement parameters (Å^2^)

**Table:**

	*U*^11^	*U*^22^	*U*^33^	*U*^12^	*U*^13^	*U*^23^
C2	0.0401 (16)	0.130 (4)	0.0470 (16)	0.021 (2)	0.0094 (13)	0.009 (2)
C3	0.0419 (15)	0.0449 (17)	0.0431 (14)	0.0097 (12)	0.0079 (11)	0.0031 (12)
C4	0.0467 (17)	0.0527 (19)	0.0437 (15)	0.0000 (14)	0.0027 (12)	−0.0069 (13)
C5	0.0312 (13)	0.0394 (16)	0.0447 (15)	0.0055 (11)	0.0040 (11)	0.0105 (12)
C6	0.0375 (13)	0.0336 (14)	0.0359 (13)	0.0016 (11)	0.0078 (10)	0.0053 (11)
C7	0.0317 (12)	0.0351 (14)	0.0394 (12)	0.0002 (12)	0.0043 (10)	0.0052 (12)
C8	0.0304 (13)	0.0492 (18)	0.0543 (16)	0.0029 (13)	0.0022 (12)	0.0009 (14)
C9	0.0375 (15)	0.0553 (19)	0.0639 (17)	0.0045 (14)	0.0122 (13)	0.0024 (15)
C10	0.0404 (14)	0.0346 (16)	0.0615 (17)	−0.0002 (12)	0.0087 (13)	−0.0024 (14)
C11	0.0378 (15)	0.062 (2)	0.0380 (14)	0.0087 (14)	0.0025 (11)	0.0069 (13)
C12	0.0437 (17)	0.095 (3)	0.074 (2)	0.0004 (19)	−0.0150 (15)	0.017 (2)
C13	0.096 (3)	0.048 (2)	0.076 (2)	0.0055 (19)	0.0225 (19)	−0.0130 (18)
Cl1	0.0594 (5)	0.0493 (5)	0.1014 (7)	0.0004 (4)	0.0246 (4)	0.0029 (5)
Cl2	0.0401 (4)	0.0873 (7)	0.1296 (8)	0.0112 (4)	0.0332 (5)	0.0213 (6)
Cl3	0.0909 (7)	0.1090 (8)	0.0525 (4)	−0.0057 (6)	0.0185 (4)	0.0003 (5)
O1	0.0543 (15)	0.161 (3)	0.0849 (18)	0.0531 (18)	0.0137 (13)	0.026 (2)
O2	0.0549 (12)	0.0344 (11)	0.0407 (10)	0.0050 (8)	0.0117 (8)	0.0045 (8)
O3	0.0771 (15)	0.0365 (12)	0.0708 (14)	0.0036 (11)	0.0056 (11)	0.0099 (11)
O4	0.0290 (9)	0.0485 (12)	0.0558 (10)	0.0062 (8)	0.0093 (7)	0.0142 (9)
O5	0.0339 (9)	0.0780 (15)	0.0454 (10)	0.0122 (10)	0.0086 (8)	0.0120 (11)
O6	0.0526 (13)	0.0589 (16)	0.0721 (14)	0.0066 (11)	−0.0053 (10)	0.0246 (12)
O7	0.0336 (9)	0.0472 (12)	0.0479 (10)	0.0013 (9)	−0.0038 (7)	0.0051 (9)
O8	0.0382 (10)	0.0553 (12)	0.0389 (9)	0.0044 (9)	0.0000 (8)	0.0068 (9)
N1	0.0384 (14)	0.083 (2)	0.095 (2)	0.0141 (15)	0.0109 (13)	0.0363 (19)
C1	0.045 (2)	0.251 (7)	0.080 (3)	−0.011 (3)	0.0175 (19)	0.045 (4)

### Geometric parameters (Å, °)

**Table:**

C2—O1	1.178 (5)	C9—Cl2	1.752 (3)
C2—O5	1.343 (4)	C9—Cl3	1.763 (3)
C2—C1	1.502 (6)	C9—Cl1	1.771 (3)
C3—O5	1.442 (3)	C10—O3	1.187 (3)
C3—C4	1.506 (4)	C10—O2	1.353 (3)
C3—C7	1.512 (4)	C10—C13	1.467 (4)
C3—H3A	0.9800	C11—O6	1.185 (4)
C4—O8	1.430 (3)	C11—O7	1.344 (3)
C4—H4A	0.9700	C11—C12	1.486 (4)
C4—H4B	0.9700	C12—H12A	0.9600
C5—O8	1.385 (3)	C12—H12B	0.9600
C5—O4	1.438 (3)	C12—H12C	0.9600
C5—C6	1.519 (3)	C13—H13A	0.9600
C5—H5A	0.9800	C13—H13B	0.9600
C6—O2	1.432 (3)	C13—H13C	0.9600
C6—C7	1.508 (4)	N1—H1	0.8820
C6—H6A	0.9800	C1—H1A	0.9600
C7—O7	1.434 (3)	C1—H1B	0.9600
C7—H7A	0.9800	C1—H1C	0.9600
C8—N1	1.234 (4)	C1—H1'1	0.9600
C8—O4	1.345 (3)	C1—H1'2	0.9600
C8—C9	1.533 (4)	C1—H1'3	0.9600
			
O1—C2—O5	124.7 (4)	O2—C10—C13	110.9 (3)
O1—C2—C1	125.7 (3)	O6—C11—O7	123.6 (3)
O5—C2—C1	109.6 (4)	O6—C11—C12	125.4 (3)
O5—C3—C4	107.0 (2)	O7—C11—C12	111.0 (3)
O5—C3—C7	109.2 (2)	C11—C12—H12A	109.5
C4—C3—C7	109.8 (2)	C11—C12—H12B	109.5
O5—C3—H3A	110.2	H12A—C12—H12B	109.5
C4—C3—H3A	110.2	C11—C12—H12C	109.5
C7—C3—H3A	110.2	H12A—C12—H12C	109.5
O8—C4—C3	113.0 (2)	H12B—C12—H12C	109.5
O8—C4—H4A	109.0	C10—C13—H13A	109.5
C3—C4—H4A	109.0	C10—C13—H13B	109.5
O8—C4—H4B	109.0	H13A—C13—H13B	109.5
C3—C4—H4B	109.0	C10—C13—H13C	109.5
H4A—C4—H4B	107.8	H13A—C13—H13C	109.5
O8—C5—O4	111.1 (2)	H13B—C13—H13C	109.5
O8—C5—C6	111.5 (2)	C10—O2—C6	116.1 (2)
O4—C5—C6	106.32 (19)	C8—O4—C5	118.1 (2)
O8—C5—H5A	109.3	C2—O5—C3	117.2 (3)
O4—C5—H5A	109.3	C11—O7—C7	117.0 (2)
C6—C5—H5A	109.3	C5—O8—C4	114.1 (2)
O2—C6—C7	107.34 (19)	C8—N1—H1	115.3
O2—C6—C5	111.3 (2)	C2—C1—H1A	109.5
C7—C6—C5	111.2 (2)	C2—C1—H1B	109.5
O2—C6—H6A	109.0	H1A—C1—H1B	109.5
C7—C6—H6A	109.0	C2—C1—H1C	109.5
C5—C6—H6A	109.0	H1A—C1—H1C	109.5
O7—C7—C6	106.3 (2)	H1B—C1—H1C	109.5
O7—C7—C3	111.17 (19)	C2—C1—H1'1	109.5
C6—C7—C3	109.9 (2)	H1A—C1—H1'1	51.8
O7—C7—H7A	109.8	H1B—C1—H1'1	140.8
C6—C7—H7A	109.8	H1C—C1—H1'1	60.7
C3—C7—H7A	109.8	C2—C1—H1'2	109.5
N1—C8—O4	124.2 (3)	H1A—C1—H1'2	60.7
N1—C8—C9	128.1 (3)	H1B—C1—H1'2	51.8
O4—C8—C9	107.8 (2)	H1C—C1—H1'2	140.8
C8—C9—Cl2	111.5 (2)	H1'1—C1—H1'2	109.5
C8—C9—Cl3	108.8 (2)	C2—C1—H1'3	109.5
Cl2—C9—Cl3	108.89 (17)	H1A—C1—H1'3	140.8
C8—C9—Cl1	109.6 (2)	H1B—C1—H1'3	60.7
Cl2—C9—Cl1	108.52 (17)	H1C—C1—H1'3	51.8
Cl3—C9—Cl1	109.53 (17)	H1'1—C1—H1'3	109.5
O3—C10—O2	122.1 (3)	H1'2—C1—H1'3	109.5
O3—C10—C13	127.0 (3)		
			
O5—C3—C4—O8	64.9 (3)	O3—C10—O2—C6	−3.8 (4)
C7—C3—C4—O8	−53.5 (3)	C13—C10—O2—C6	176.7 (2)
O8—C5—C6—O2	174.5 (2)	C7—C6—O2—C10	−159.8 (2)
O4—C5—C6—O2	53.3 (3)	C5—C6—O2—C10	78.3 (3)
O8—C5—C6—C7	54.9 (3)	N1—C8—O4—C5	−3.4 (4)
O4—C5—C6—C7	−66.4 (2)	C9—C8—O4—C5	175.5 (2)
O2—C6—C7—O7	64.1 (2)	O8—C5—O4—C8	98.2 (3)
C5—C6—C7—O7	−173.92 (18)	C6—C5—O4—C8	−140.3 (2)
O2—C6—C7—C3	−175.5 (2)	O1—C2—O5—C3	−2.1 (5)
C5—C6—C7—C3	−53.6 (3)	C1—C2—O5—C3	177.3 (3)
O5—C3—C7—O7	52.8 (3)	C4—C3—O5—C2	146.6 (3)
C4—C3—C7—O7	169.9 (2)	C7—C3—O5—C2	−94.6 (3)
O5—C3—C7—C6	−64.5 (3)	O6—C11—O7—C7	1.3 (4)
C4—C3—C7—C6	52.5 (3)	C12—C11—O7—C7	−178.5 (2)
N1—C8—C9—Cl2	−3.6 (4)	C6—C7—O7—C11	−155.7 (2)
O4—C8—C9—Cl2	177.5 (2)	C3—C7—O7—C11	84.8 (3)
N1—C8—C9—Cl3	116.5 (3)	O4—C5—O8—C4	62.6 (3)
O4—C8—C9—Cl3	−62.4 (3)	C6—C5—O8—C4	−55.8 (3)
N1—C8—C9—Cl1	−123.8 (3)	C3—C4—O8—C5	56.4 (3)
O4—C8—C9—Cl1	57.3 (3)		

### Hydrogen-bond geometry (Å, °)

**Table:**

*D*—H···*A*	*D*—H	H···*A*	*D*···*A*	*D*—H···*A*
N1—H1···Cl2	0.88	2.55	3.009 (3)	114
